# Definitions of early COPD and markers and tools for predicting progression: a systematic review protocol

**DOI:** 10.1136/bmjopen-2025-100032

**Published:** 2025-06-03

**Authors:** Alastair Watson, Gbenga Adesoye, Kane Alexander, Ross Davidson, Fu Chuen Kon, Arnav Sharma, Mosea Song, Isobel Soper, Akhilesh Jha, Marie Fisk

**Affiliations:** 1 University of Cambridge School of Clinical Medicine, Cambridge, UK; 2 Cambridge University Hospitals NHS Foundation Trust, Cambridge, UK; 3 Chelsea and Westminster Hospital NHS Foundation Trust, London, UK; 4 Royal Free London NHS Foundation Trust, London, UK

**Keywords:** Systematic Review, Lung Diseases, RESPIRATORY MEDICINE (see Thoracic Medicine), Adult thoracic medicine, Chronic airways disease

## Abstract

**Abstract:**

**Introduction:**

Chronic obstructive pulmonary disease (COPD) is a leading cause of morbidity and mortality globally. Early diagnosis and intervention are crucial for preventing disease progression. The concept of early COPD is considered to represent the initial phase of the disease course. However, different terms are used, and a standardised definition is lacking. This has hindered research and clinical utility. This systematic review aims first to examine current early COPD research and outline the definitions and terms used to help reach consensus and direct future clinical research. Second, it will identify currently proposed markers and tools for predicting the progression of early COPD and the quality of evidence to help direct future research and facilitate the development of novel management strategies.

**Methods and analysis:**

This study will search for all clinical studies on early COPD, using a standardised search strategy, searching CENTRAL (the Cochrane Library), MEDLINE (Ovid), PubMed, Scopus, Web of Science and Google Scholar. Titles and abstracts will be reviewed and compared against inclusion and exclusion criteria. Stage 1 of this review will assess the terms and definitions used for early COPD. Stage 2 will assess studies presenting additional markers or tools for predicting the progression of early COPD. Study quality will be assessed using a modified Downs and Black checklist for observational studies and the risk of bias (RoB) 2 tool for randomised controlled trials. This protocol has been registered in PROSPERO (CRD42025645320).

**Ethics and dissemination:**

This systematic review will use freely available data within the literature and will not directly involve human participants; therefore, ethical approval is not required. The results of this systematic review will be prepared and submitted for presentation as conference presentation(s) and for publication as a peer-reviewed article.

**PROSPERO registration number:**

CRD42025645320.

**STRENGTHS AND LIMITATIONS OF THIS STUDY**

- PROSPERO registration: (CRD42025645320) ensures protocol preregistration, reducing the risk of bias.
- Comprehensive scope: inclusion of all early chronic obstructive pulmonary disease (COPD) synonyms and definitions (including early COPD, GOLD 0 (defined as the presence of risk factors (smoking) and symptoms (chronic cough and sputum production) in the absence of spirometric abnormalities that cross the diagnostic threshold for COPD), preserved ratio impaired spirometry, pre-COPD and at-risk COPD) to capture broad evidence.
- Patient and public involvement: engaged with patients with COPD prior to writing the protocol to ensure the review aligns with the needs and perspectives of patients.
- Inclusion of all early COPD definitions and types of markers: may limit data synthesis and interpretation due to heterogeneity.
- Reliance on published markers and tools: may exclude emerging or unpublished predictive methods and limit insights into novel predictive tools under development.

## Introduction

 Chronic obstructive pulmonary disease (COPD) remains a significant global health emergency, contributing substantially to morbidity and mortality despite the existence of current treatment strategies and evidence-based guidelines.^1^,^5^ This heterogeneous disease encompasses a range of pathological processes, manifesting in different pathological entities, including emphysema, chronic bronchitis and small airway diseases, which can coexist with varying degrees of severity and progress at different rates among individuals.

Early diagnosis of COPD is crucial as it allows the implementation of preventative and therapeutic strategies to slow disease progression and prevent the development of more complex established disease states, associated with increased morbidity and mortality.^6^ Early intervention also presents opportunities for the use of future targeted therapeutics earlier in the disease history, when disease processes are more tractable to treatment. To achieve this, numerous initiatives have been implemented and are currently underway to facilitate earlier diagnosis.^7^,^9^

Thus, there has been growing interest in the concept of ‘early COPD’.^10^,^12^ Early COPD is difficult to define, and its association with disease activity is also uncertain and likely varies across different individuals. Early COPD is considered to represent the initial phase of the disease course rather than indicating its severity or potential future severity. Therefore, it is important to note that early COPD is not synonymous with ‘mild COPD’, yet some clinical trials have used these terms interchangeably, contributing to confusion in the literature.^13^ A consensus on the definition of early COPD has not yet been established. This has limited the progression of this field and its operational use clinically. Recently, two definitions have been proposed by Martinez *et al*^11^ and Siafakas *et al*.^14^ However, other definitions continue to be employed, incorporating variables around spirometry measures and their temporal changes, symptoms, functional small airway disease and other CT findings.^13^ Thus, interpreting and progressing the current evidence base around early COPD has been challenging, and the opportunity to use this term as a clinical entity has been limited. This has been further complicated by the use of overlapping and synonymous terms such as pre-COPD, at-risk COPD, preclinical COPD, undiagnosed COPD, GOLD stage 0 and preserved ratio impaired spirometry (PRISm). Furthermore, GOLD 2025 COPD guidance more recently advises using the term to encompass the ‘biological’ first steps of the disease in an experimental setting. What this means practically is uncertain.^5 11^

Understanding which terms are used in the literature and their evidence base, and achieving a consensus on definition, is essential for the development, testing and implementation of individualised interventions to prevent the development of irreversible disease mechanisms and disease progression. Furthermore, understanding the quality of current evidence in the field of early COPD is essential to direct future research.

In this systematic review, we aim to comprehensively review the current clinical research on early COPD, understand which terms are used and how they are defined, with a focus on variables that are included in the proposed working definitions. A thorough examination of the current definitions is essential to facilitate their standardisation for research and clinical use. We further propose to review the published literature on markers and tools for predicting the progression of early COPD to help facilitate their further development and validation. This review will aid future research and development of individualised interventions based on COPD disease trajectory, which is a crucial part of the strategies needed to address the unmet need of COPD.^15^

## Objectives

We aim to systematically review the literature on early COPD to assess:

- What terms are used to refer to early COPD, and how these terms are defined?
- What tools have been developed and tested to predict the progression of early COPD, how effective they are and what is the quality of the evidence?

Through this systematic review, we aim to provide insights into the consistency and quality of early COPD research to help reach a consensus, allow the use of this concept in clinical practice and inform future studies to understand how to predict disease trajectories in patients with early COPD.

## Methods and analysis

This systematic review protocol was registered on PROSPERO (CRD42025645320) and written with reference to the Cochrane methodology,^16^ Joanna Briggs Institute (JBI) manual^17^ and Preferred Reporting Items for Systematic Review and Meta-Analysis Protocols (PRISMA-P, 2015 guidelines). The review will include a PRISMA flow chart with the full process.

### Search strategy

Three independent researchers designed the search strategy. They agreed on the following search terms: *pre-COPD*; *preCOPD; preclinical COPD*; *prodromal COPD*; *at-risk COPD*; *incipient COPD*; *early COPD*; *early-stage COPD*; undiagnosed COPD; *GOLD 0*, *Preserved Ratio Impaired, PRISm* OR *early diagnosis* (*combined with COPD* OR *chronic obstructive pulmonary disease*).

We will perform the search in February 2025 using the following databases: CENTRAL (the Cochrane Library), MEDLINE (Ovid), PubMed, Scopus and Web of Science for articles using our proposed search. All published material with these keywords in the abstract or title will be included in the search. An additional search will be performed using Google Scholar (online supplemental material), and the first 1000 articles (ordered by relevance) will be reviewed by title/abstract for consideration of inclusion. The full search strategies and searches to be used for each database are given in the online supplemental material.

The search will be comprehensive, with no restrictions on publication year (all publications up to the date of the search will be included), language or publication type to ensure that relevant studies are not missed. Non-English studies will be translated using Google Translate.^18^ The ability of translated results to be adequately interpreted will be assessed by two independent reviewers. If the translated article is deemed not able to be interpreted sufficiently, it will be excluded. Disagreements will be resolved through discussion. Relevant abstracts will be carefully evaluated, and corresponding authors will be contacted to obtain access to any grey literature or additional materials or information, as needed. References of key articles and key review articles will also be reviewed to identify any additional publications not already identified.

### Selection process

Search results will be manually reviewed initially by two individual reviewers, and those pertaining to a human clinical study will be taken forward for consideration of selection. At least two reviewers will independently evaluate the title and abstract of all relevant articles and compare them against the inclusion and exclusion criteria (table 1). If inconsistencies occur, a consensus will be reached through discussion or, if this is not possible, then the decision will be made by an additional reviewer. Endnote, Word, Excel or libraries of the online databases will be used to track references.

**Table 1:** Inclusion and exclusion criteria

Stage 1: terms used for early COPD and their definitions	
Inclusion criteria	Exclusion criteria
Studies that have a defined population of participants with features of early COPD, aim to identify participants with early COPD or one of the following (or other) synonyms of early COPD: pre-COPD; preclinical COPD; prodromal COPD; at-risk COPD; incipient COPD; early-stage COPD; GOLD 0 or preserved ratio impaired spirometry (PRISm), where a definition is given or where the definition or criteria can be discerned	Studies involving only patients with an established diagnosis of COPD according to the GOLD definition or patients identified to have COPD as per the GOLD criteria within the study. This includes studies that are case-finding for previously undiagnosed established COPD disease. This also includes studies that label participants with early COPD who have an established COPD diagnosis or that would meet the GOLD grade of COPD I–IV, without a clear rationale as to why they are considered early COPD or at the early stage of their disease, rather than being patients with mild or moderate COPD*
Human clinical study	Publication not involving a human clinical study
Studies published as full original articles, research letters or abstracts with sufficient information about how they define a population with early COPD or one of the above synonyms	Other article types, such as review articles (including systematic reviews), case reports, editorials, protocols, trial registrations or other publications without original data. Or studies published only as abstracts without sufficient information about how they define a population with early COPD or one of the above synonyms
	Studies that do not include defined criteria of early COPD (or other similar terms) or where the criteria are not able to be discerned
	Articles not able to be accessed by the team, or not available after contacting the corresponding author, and where there is not sufficient information within the abstract about their criteria for early COPD or one of the above synonyms
**Additional criteria for stage 2: markers or tools for predicting progression of early COPD**	
Studies published as full original articles	Studies published as other article types, including abstracts, research letters and those listed above
Human clinical studies that contain markers, biomarkers or prediction tools which could be used for or suggested as having the potential to predict the progression of early COPD	Studies that do not include markers, biomarkers or prediction tools which could be used for or are suggested as having the potential to predict the progression of early COPD
Longitudinal studies that assess progression from baseline to follow-up	Studies that are cross-sectional in nature, have another design that is not longitudinal or present only associations based on one timepoint
Studies which compare patients with early COPD who progress versus those who do not using quantifiable outcomes (including ORs/relative risks and area under the curve)	Studies that only include markers, biomarkers or tools which are not shown to be useful/suggested to be useful or have potential use for predicting the progression of early COPD or do not report quantifiable outcomes in written format
	Articles not able to be accessed by the team or are not available after contacting the corresponding author

* Although studies where patients with mild/moderate COPD are labelled as having early COPD will be excluded from this review, the number of these studies that do this will be recorded and presented in the results.

COPD, chronic obstructive pulmonary disease.

### Overview of systematic review design

This study will contain two stages. The first stage will identify all clinical studies which include participants with early COPD (or similar terms) and the definitions that they use. This aims to understand the terms used in the current literature base and how they are defined. These studies will then be assessed for their suitability for inclusion in the second stage, which will identify studies with relevant markers, biomarkers or prediction tools which have the potential or are suggested to have potential for predicting the progression of early COPD.

### Outcome measurements

The primary outcomes will be:

1. The frequency of each of the different terms used for early COPD.
2. Definitions used in the study of early COPD. Specifically, are the following (taken from definitions proposed by Martinez *et al*^11^ or Siafakas *et al*^14^) met?
  - Does the study include the following individual parameters in their inclusion criteria?
    - Age≤50 years.
    - Smoking history of ≥10 pack-years.
    - Spirometry criteria of forced expiratory volume in 1 s (FEV1)/forced vital capacity (FVC)<lower limit of normal (LLN).
    - Spirometry criteria of FEV1/FVC >0.7.
    - Spirometry criteria FEV1>80% predicted.
    - CT abnormalities criterion: inclusion of compatible CT abnormalities.
    - Accelerated lung function decline, defined by a reduction of FEV1 by at least 60 mL or more per year.
    - Compatible respiratory symptoms.
  - Are any other criteria included within the definitions used in the study, and if so, what are they?
  - Does the study’s inclusion criteria meet the Martinez *et al* inclusion criteria of age ≤50 years, a smoking history of at least 10 pack years and any one of the following: FEV1/FVC less than LLN, compatible CT abnormalities or accelerated FEV1 decline of at least 60 mL/year?
  - Does the study’s inclusion criteria meet the Siafakas *et al* inclusion criteria of FEV1/FVC >0.7, FEV1>80% predicted and the presence of compatible respiratory symptoms?
3. What markers, biomarkers or prediction tools have been proposed for predicting the progression of early COPD, and how sensitive/specific are they?
4. What is the risk of bias/quality of the studies on markers, biomarkers or prediction tools?

### Inclusion and exclusion criteria

Table 1 presents the inclusion and exclusion criteria for stage 1 of this systematic review and the additional inclusion and exclusion criteria for stage 2.

### Data management and extraction

Data regarding study characteristics (author, year, study design and study setting), inclusion criteria (as detailed above), biomarkers, prediction tools and outcomes will be extracted and recorded in a database. Data on variables within manuscripts will also be assessed by a second independent reviewer, and the accuracy of the initial extraction will be reviewed. Any discrepancies or errors will be resolved through discussion and, where relevant, correction. Variables that will be extracted in stage 2 will include: term(s) used; definitions of terms; study name (where relevant); country; year of enrolment; study design; length of follow-up; the number of participants; average age; sex distribution; ethnicity; body mass index; baseline COPD symptoms; smoking status; pack year history; frequency of other exposures (eg, occupational/secondhand smoke); baseline spirometry ranges; baseline rates of lung function decline at recruitment; baseline CT abnormalities; definition of disease progression; type of marker and details of marker; use of a validation cohort or not and effect measures and results. Data will be summarised and presented with summary statistics where appropriate. It is unknown at this point whether the data will be suitable for quantitative analysis. However, if the data are suitable for quantitative analysis and the same or comparable units of variables or simple validated conversion of units, then the data will be considered for combining and performing a meta-analysis. Variability of the data, if such analysis is undertaken, will be assessed by I^2^ statistics. A further statistical analysis plan will be formulated once the data have been collected, if required.

### Critical appraisal of studies

The methodological quality of all included studies will be assessed by at least two independent reviewers. Observational studies will be appraised using a modified Downs and Black checklist (online supplemental material). This was modified to encompass only questions related to cohort studies specifically. Questions relating to randomised controlled trials (RCTs), including adverse events, attempts to blind the study, compliance to the intervention and randomisation, were excluded. RCTs will be assessed using the RoB 2 tool.^19^

### Interpretation of findings

The Grading of Recommendation Assessment, Development and Evaluation (GRADE) will be used to assess evidence quality and strength.

### Patient and public involvement

We engaged with five COPD patients, distributing a questionnaire with a lay summary of the study to gather their insights on our systematic review (online supplemental material). We provided a lay summary of our review and sought their feedback on its perceived usefulness and answered any questions they may have, with an aim to identify any considerations which had not previously been discussed by the review team. We aimed to elicit any other considerations they believed we should address in this or future studies. We received four responses. This input helped us ensure that our review aligns with the needs and perspectives of patients.

## Ethics and dissemination

This protocol and systematic review will use data freely available within the literature and will not directly involve human participants; ethical approval is therefore not required. The results of this systematic review will be written up and submitted for presentation as conference presentation(s) and for publication as a peer-reviewed article.

## Supplementary material

10.1136/bmjopen-2025-100032 online supplemental file 1
