# Supplementary material for: Definitions of early COPD and markers and tools for predicting progression: a systematic review protocol
Source: BMJ Open. 2025 Jun 3;15(6):e100032. doi: 10.1136/bmjopen-2025-100032 (PMC12142007; doi:10.1136/bmjopen-2025-100032)
Supplement: online supplemental file 1 [file bmjopen-15-6-s001.docx]

# Supplementary material: Definitions of early COPD and markers and tools for predicting progression: a systematic review protocol

## Public and Patient Involvement Questionnaire

*We want your help to plan our research. Below we have written a summary of our planned research and your opinions on it would be very helpful.*

**Summary**

Chronic Obstructive Pulmonary Disease or ‘COPD’ for short; is a lung condition that affects millions of people in the UK and worldwide. People living with COPD often have symptoms of breathlessness and cough and get flare-ups of their COPD.

A current problem with treating COPD, is that often people have long-term lung impairment by the time they are diagnosed with COPD.

Some research work is underway to make it easier to spot people who may be *at risk of developing* COPD, in order to intervene earlier and help avoid long-term lung problems before they take hold. These patients are said to have "early COPD". But there's a problem: people can't agree on what early COPD really means. Different doctors use different definitions. This makes it hard to study how to find patients with early COPD.

To fix this, we want to look at all the information we have about how people define ‘early COPD’. We hope this will help everyone agree on one definition. We also want to look at ways people have suggested to spot patients with early COPD and those who might get worse. We hope this will help us to find better ways to help people who may be at risk of developing COPD sooner.

**Questions**

We wanted to get your opinion on this research, including:

1.  What are your general thoughts about this work?

2.  Do you think this will be important for patients with COPD?

3.  Are there any things you feel we have not considered?

4.  Is there anything else we should consider in the future related to this research?

5.  Do you think defining "early COPD" is important? Why or why not?

7.  How do you think doctors could better identify people at risk of developing COPD?

8. Are there any symptoms or experiences you had for a while before your COPD diagnosis that you think doctors should have paid more attention to?

9. How do you think your treatment might have been different if you were diagnosed earlier

10. What barriers do you think exist for people getting help with a lung health check sooner?

## Search strategies

Below are the searches which we designed for use for each database. To ensure that articles catalogued with incorrect filters were not missed, and to overcome problems identified in the preliminary searches of not returning specific articles of relevance, no specific filters were used, except filtering for the search terms to be contained within the title or abstract. This included no filters being used based on article type or study type. The following search was used in Pubmed CENTRAL (the Cochrane Library), MEDLINE (Ovid), PubMed, Scopus, Web of Science filtering based on terms appearing in either the abstract or title:

"pre-COPD" OR "preCOPD" OR "preclinical COPD" OR "prodromal COPD" OR "at-risk COPD" OR "incipient COPD" OR "early COPD" OR "early-stage COPD" OR ("GOLD 0" AND (COPD OR "chronic obstructive pulmonary disease")) OR ("Preserved Ratio Impaired Spirometry" AND (COPD OR "chronic obstructive pulmonary disease")) OR (PRISm AND (COPD OR "chronic obstructive pulmonary disease")) OR ("early diagnosis" AND (COPD OR "chronic obstructive pulmonary disease")) OR "undiagnosed COPD

Systematic reviews often develop different searches for each database as the search syntax varies between them. However, to facilitate the use of our common search, we developed a broad search strategy which included different terms, synonyms, variants and truncations to minimise the chance of articles being missed. We further developed the strategy to search title and abstract without the use of any filters, which also allowed our search to be used across different platforms. We performed preliminary searches to check the utility of the search in each database and amended it iteratively, until the final search (above) was developed. We further analysed the results from each platform search against a list of key articles from the grey literature to ensure that these articles were returned. Having taken these steps to ensure the rigour of our search strategy, we chose to use the above search across all the core databases included within our study.

The following search was also done in Google scholar using the following in the free text box: "pre-COPD" OR "preCOPD" OR "preclinical COPD" OR "prodromal COPD" OR "at-risk COPD" OR "incipient COPD" OR "early COPD" OR "early-stage COPD" OR ("GOLD 0" AND (COPD OR "chronic obstructive pulmonary disease")) OR ("Preserved Ratio Impaired Spirometry" AND (COPD OR "chronic obstructive pulmonary disease")) OR (PRISm AND (COPD OR "chronic obstructive pulmonary disease")) OR ("early diagnosis" AND (COPD OR "chronic obstructive pulmonary disease")) OR "undiagnosed COPD".

## Modified Downs and Blacks

Reporting

**1. Is the hypothesis/ aim/ objective of the study clearly described? (yes = 1, no = 0)**

**2. Are the main outcomes to be measured clearly described in the introduction or methods section? (yes = 1, no = 0)** If the main outcomes are first mentioned in the Results section, the question should be answered no.

**3. Are the characteristics of the patients included in the study clearly described? (yes = 1, no = 0)** Inclusion/ exclusion criteria given in cohort studies

**4. Are the exposures/predictor variables of interest clearly described? (yes = 1, no = 0)**

**5. Are the distributions of principal confounders in each group of subjects to be compared clearly described? (yes = 2, partially = 1, no = 0)** Is a list of all principal confounders provided? For the sake of clarity, this should be defined prior to commencement of the checklist.

**6. Are the main findings of the study clearly described? (yes = 1, no = 0)** Simple outcome data (including denominators and numerators) should be reported for all major findings so that the reader can check the major analyses and conclusions. (This question does not cover statistical tests which are considered later)

**7. Does the study provide estimates of the random variability in the data for the main outcomes? (yes = 1, no = 0)** In non-normally distributed data the inter-quartile range of results should be reported. In normally distributed data the standard error, standard deviation or confidence intervals should be reported. If the distribution of the data is not described, it must be assumed that the estimates used were appropriate and the question should be answered yes.

**8. Have the characteristics of patients lost to follow-up been described? (yes = 1, no = 0)** This should be answered yes where there were no losses to follow-up or where losses to follow-up were so small that findings would be unaffected by their inclusion. This should be answered no where a study does not report the number of patients lost to follow-up.

**9. Have actual probability values been reported (e.g. P = 0.035 rather than <0.05) for the main outcomes except where the probability value is less than 0.001? (yes = 1, no = 0)**

External Validity

**10. Were the subjects asked to participate in the study representative of the entire population from which they were recruited? (yes = 1, no = 0, unable to determine = 0)** The study must identify the source population for patients and describe how the patients were selected. Patients would be representative if they comprised the entire source population, an unselected sample of consecutive patients, or a random sample. Random sampling is only feasible where a list of all members of the relevant population exists. Where a study does not report the proportion of the source population from which patients are derived, the question should be answered as unable to determine.

**11. Were those subjects who were prepared to participate representatives of the entire population from which they were recruited? (yes = 1, no = 0, unable to determine = 0)** The proportion of those asked who agreed should be stated. Validation that the sample was representative would include demonstrating that the distribution of the main confounding factors was the same in the study sample and the source population.

**12 Were the settings, staff, and facilities where participants were assessed representative of those available to the majority of the source population?.** The question should be answered no if, for example, the intervention was undertaken in a specialist centre unrepresentative of the hospitals most of the source population would attend, or the intervention utilizes specialist staff or equipment not normally available (e.g. training using a Biodex).

Internal validity – bias

**13. If any of the results of the study were based on "data dredging", was this made clear? (yes = 1, no = 0, unable to determine = 0)** Any analyses that had not been planned at the outset of the study should be clearly indicated. If no retrospective unplanned subgroup analyses were reported, then answer yes.

**14. Do the analyses adjust for different lengths of follow-up of patients ? (yes = 1, no = 0, unable to determine = 0)** Where follow-up was the same for all study patients the answer should be yes. If different lengths of follow-up were adjusted for by, for example, survival analysis, the answer should be yes. Studies where differences in follow-up are ignored should be answered no. Where there was no follow up period after the intervention, this should be answered no.

**15 Were the statistical tests used to assess the main outcomes appropriate? (yes = 1, no = 0, unable to determine = 0**) The statistical techniques used must be appropriate to the data. For example non-parametric methods should be used for small sample sizes. Where little statistical analysis has been undertaken but where there is no evidence of bias, the question should be answered yes. If the distribution of the data (normal or not) is not described it must be assumed that the estimates used were appropriate and the question should be answered yes.

**16. Were the main outcome measures used accurate (valid & reliable)? (yes = 1, no = 0, unable to determine = 0)** For studies where the outcome measures are clearly described, the question should be answered yes. For studies which refer to other work or that demonstrates the outcome measures are accurate, the question should be answered as yes.

Internal validity – confounding (selection bias)

**17. Were the patients in different intervention groups (trial & cohort studies) or were the cases & controls (case-control studies) recruited from the same population? (yes = 1, no = 0, unable to determine = 0)** For example, patients for all comparison groups should be selected from the same hospital. The question should be answered unable to determine for cohort and case control studies where there is no information concerning the source of patients included in the study.

**18. Were study subjects in different intervention groups (trials & cohort studies) or were the cases & controls (case-control studies) recruited over the same period of time? (yes = 1, no =0, unable to determine = 0)** Where this is demonstrated in a flow-chart or diagram, this should be answered yes. For a study which does not specify the time period over which patients were recruited, the question should be answered as unable to determine.

**19. Was there adequate adjustment for confounding in the analyses from which the main findings were drawn? (yes = 1, no = 0, unable to determine = 0)** In non-randomised studies if the effect of the main confounders was not investigated or confounding was demonstrated but no adjustment was made in the final analyses, the question should be answered as no.

**20. Were losses of patients to follow-up taken into account? (yes = 1, no = 0, unable to determine = 0)** If the numbers of patients lost to follow-up are not reported, the question should be answered as unable to determine. If the proportion lost to follow-up was too small to affect the main findings, the question should be answered yes.

Power

**21. Did the study have sufficient power to detect a clinically important effect where the probability value for a difference being due to chance is less than 5%? (yes = 1, no = 0)** This should be answered as yes where a study has performed a power calculation and the required sample size was met. Where the required sample size was not met, or where no power calculation was performed, this should be answered no.

**To discuss clear discrepancies and agree**
(Out of 22)
