# Supplementary material for: Definitions of early COPD and markers and tools for predicting progression: a systematic review protocol
Source: BMJ Open. 2025 Jun 3;15(6):e100032. doi: 10.1136/bmjopen-2025-100032 (PMC12142007; doi:10.1136/bmjopen-2025-100032)
Supplement: Supplementary file 2 [file bmjopen-2025-100032.reviewer_comments.pdf]

PEER REVIEW HISTORY

BMJ Open publishes all reviews undertaken for accepted manuscripts. Reviewers are asked to complete a checklist review form and are provided with free text boxes to elaborate on their assessment. These free text comments are reproduced below.

ARTICLE DETAILS

Title (Provisional)

Definitions of early COPD and markers and tools for predicting progression: a systematic review protocol

Authors

Watson, Alastair; Adesoye, Gbenga; Alexander, Kane; Davidson, Ross; Kon, Fu; Sharma, Arnav; Song, Mosea; Soper, Isobel; Jha, Akhilesh; Fisk, Marie

---

VERSION 1 - REVIEW

**Reviewer**	**1**
**Name**	**Welch, Lindsay**
**Affiliation**	**Bournemouth University**
**Date**	**26-Mar-2025**
**COI**	**None**

This is a needed review to understand the nature and definition of early COPD. I have some comments to strengthen the protocol.

Early COPD – concept

This is needed research – the concept of early COPD, or early changes in the airways or symptoms suggestive of COPD is not well defined.

The methods are well described.

Drawing evidence from the literature in terms of nomenclature and also biomarkers as clinical indicators.

Ideally, they need to be registered in PROSPERO before starting – note this is the protocol.

Also Joanna Briggs institute guidance on systematic reviews would have strengthened the methodology JBI Evidence Implementation although noted the proposal.

Consider including all language studies- how will you translate studies if working in English.

**Reviewer**	**2**
**Name**	**Cherneva, R**

**Affiliation**	**University Hospital Alexandrovska**
**Date**	**22-Apr-2025**
**COI**	**None**

I think that the idea of the review is original, since it is not so commonly discussed. It has two aspects - one clinical and one one that is more associated with the basic science - biomarkers. I recommend the publication of the manuscript with minor revisions. I think the schematic design makes it easier for the reader to comprehend the idea.

VERSION 1 - AUTHOR RESPONSE

Reviewer Comments:

Reviewer 1: This is a needed review to understand the nature and definition of early COPD. I have some comments to strengthen the protocol.

Authors: We thank the reviewer for their appreciation of the importance of this review. We have made all requested changes and agree that this strengthens the protocol.

Responses to comments in the PDF 'Review Early COPD':

BMJ review

Early COPD – concept

Reviewer: This is needed research – the concept of early COPD, or early changes in the airways or symptoms suggestive of COPD is not well defined.

Authors: We are pleased that the reviewer agrees about the importance of understanding current early COPD definitions and we feel it will take the field forward.

Reviewer: The methods are well described.

Authors: We are happy the reviewer feels that the methods are well described. We have added even further clarity in line with the editor and reviewer's suggestion.

Reviewer: Drawing evidence from the literature in terms of nomenclature and also biomarkers as clinical indicators

Authors: We feel this is an important focus of our review and that it will help direct future research.

Reviewer: Ideally, they need to be registered in PROSPERO before starting – note this is the protocol.

Authors: We agree about the importance of registering this protocol on PROSPERO to enhance transparency and minimise risk of bias. We have therefore now registered on PROSPERO (CRD42025645320) and included the registration number at the end of the abstract and in the Methods and Analysis section.

Reviewer: Also Joanna Briggs institute guidance on systematic reviews would have strengthened the methodology JBI Evidence Implementation although noted the proposal.

Authors: We thank the review for this useful comment. We have now reviewed the JBI Manual for Evidence Synthesis (2024 edition) (Reference below). On reflection of page 277 of the manual we felt the need to give detail to what type of data we plan to extract within stage 2 of our review which focuses on markers and tools for predicting progression. We have therefore edited our methods in line with this, lines 197-210 now read as

"Variables that will be extracted in stage 2 will include: terms(s) used; definitions of terms; study name (where relevant); country; year of enrolment; study design; length of follow-up; number of participants; average age; sex distribution; ethnicity; breakdown; Body mass index (BMI); baseline COPD symptoms; smoking status; pack year history; frequency of other exposures (e.g occupational/second hand smoke); baseline spirometry ranges; baseline rates of lung function decline at recruitment; baseline computed tomography (CT) abnormalities; definition of disease progression; type of marker and details of marker; use of a validation cohort or not; and effect measures and results. Data will be summarised and presented with summary statistics where appropriate. It is unknown at this point whether the data will be suitable for quantitative analysis. However, If the data is suitable for quantitative analysis and the same or comparable units of variables or simple validated conversion of units, then the data will be considered for combining and performing a meta-analysis. Variability of the data, if such analysis is undertaken, will be assessed by I2 statistics. A further statistical analysis plan will be formulated once the data has been collected, if required."

We have also explicitly compared our protocol with the JBI checklist and feel that it does adhere to this. However, we felt that in order for the protocol to fully meet point 7 of the checklist "Were there methods to minimize errors in data extraction?" we should add additional clarification to our methods section of the protocol, lines 195-197 now read as:

"Data of variables within manuscripts will also be assessed by a second independent person and the accuracy of the initial extraction will be reviewed and any discrepancies or errors will be resolved by discussion and where relevant correction."

Overall, we found this suggestion extremely useful and will use the JBI manuscript when writing up the results.

Reference: Aromataris E, Lockwood C, Porritt K, Pilla B, Jordan Z, editors. JBI Manual for Evidence Synthesis. JBI; 2024. Available from: https://synthesismanual.jbi.global

Reviewer: Consider including all language studies- how will you translate studies if working in English.

Authors: We thank you for this comment. We have carefully considered the inclusion of all language studies and feel that exclusion of non-English studies would create an inherent bias within our review. We also recognise the potential pitfalls in relying on translated versions. We have therefore added further clarification to the methods section to add transparency about these considerations and our plans to deal with this. Lines 137-142 of our methods section now reads as:

"The search will be comprehensive, with no restrictions on publication year (all publications up to date of search will be included) or language or publication type, to ensure that relevant studies are not missed. Non-English studies will be translated using Google Translate (17). The ability for translated results to be adequately interpreted will be assessed by two independent people. If the translated article is deemed not able to be interpreted sufficiently it will be excluded, disagreements will be resolved by discussion."

Reviewer 2: I think that the idea of the review is original, since it is not so commonly discussed. It has two aspects - one clinical and one one that is more associated with the basic science - biomarkers. I recommend the publication of the manuscript with minor revisions. I think the schematic design makes it easier for the reader to comprehend the idea.

Authors: We are glad that the reviewer sees the originality of our review and recommend publication. We have made all changes point by point below and hope they agree the manuscript is improved and now ready for publication.
